# Genetic Stratification in Myeloid Diseases: From Risk Assessment to Clinical Decision Support Tool

**DOI:** 10.5041/RMMJ.10159

**Published:** 2014-10-29

**Authors:** Yishai Ofran

**Affiliations:** 1 Department of Hematology and Bone Marrow Transplantation, Rambam Health Care Campus, Haifa, Israel and; 2 Bruce Rappaport Faculty of Medicine, Technion, Israel Institute of Technology, Haifa, Israel

**Keywords:** Acute myeloid leukemia, allogeneic hematopoietic cell transplantation, genetic aberrations, minimal residual disease, myelodysplastic syndrome

## Abstract

Genetic aberrations have become a dominant factor in the stratification of myeloid malignancies. Cytogenetic and a few mutation studies are the backbone of risk assessment models of myeloid malignancies which are a major consideration in clinical decisions, especially patient assignment for allogeneic stem cell transplantation. Progress in our understanding of the genetic basis of the pathogenesis of myeloid malignancies and the growing capabilities of mass sequencing may add new roles for the clinical usage of genetic data. A few recently identified mutations recognized to be associated with specific diseases or clinical scenarios may soon become part of the diagnostic criteria of such conditions. Mutational studies may also advance our capabilities for a more efficient patient selection process, assigning the most effective therapy at the best timing for each patient. The clinical utility of genetic data is anticipated to advance further with the adoption of deep sequencing and next-generation sequencing techniques. We herein suggest some future potential applications of sequential genetic data to identify pending deteriorations at time points which are the best for aggressive interventions such as allogeneic stem cell transplantation. Genetics is moving from being mostly a prognostic factor to becoming a multitasking decision support tool for hematologists. Physicians must pay attention to advances in molecular hematology as it will soon be accessible and influential for most of our patients.

## INTRODUCTION

Forty-one years ago Janet Rowley was the first to describe chromosomal translocation in leukemia.^1^ Back in the 1970s, the concept that genetic aberrations may be involved in cancer pathobiology was so revolutionary that her landmark report of t(8:21) in acute myeloid leukemia (AML) was rejected by the *New England Journal of Medicine* because reviewers thought her findings were not important.^2^ Soon afterwards, the value of her findings was recognized, and her second report regarding t(9:22) in chronic myeloid leukemia (CML) was accepted by *Nature*.^3^ During the last decades, scientists, oncologists, and hematologists have come a long way advancing our knowledge and understanding of the roles of genetic aberrations in cancer pathobiology. Numerous genetic aberrations were identified in each cancerous disease. A current clinical challenge is to pick up aberrations that matter out of a huge haystack of genetic information. Doctors and health care providers are under continuous pressure to catch up with cutting-edge discoveries. The enormous number of possible combinations of coexisting mutations and the growing number of novel available therapies make clinical implications of genetic data complicated. As in many other fields of life, the public is a step ahead of governmental or authorized health care organizations. It is common that patients initiate additional comprehensive genetic testing, expecting their treating physicians to be equipped enough to interpret such results. Unfortunately, most identified mutations are either clinically meaningless, or their value in each clinical setting is still to be recognized. In the following review an up-to-date comprehensive approach for genetic stratification in myeloid diseases will be discussed in light of common clinical dilemmas.

## GENETIC CONSIDERATIONS IN THE EVALUATION OF MYELOID DISEASES

### Genetics as a Supportive Tool for Accurate Diagnosis of Myeloproliferative Disorders, Myelodysplasia, and Neoplasms

Back in 1953 a pre-leukemic state was described in a series of 12 patients,^4^ yet a clear pathological definition, classification, and naming the syndrome as myelodysplastic were established only in 1982 by the French–American–British (FAB) group.^5^ During the 32 years that followed the FAB classification, myelodysplastic syndrome (MDS) was reclassified twice,^6^,^7^ based on morphology. Currently isolated deletion of 5q is the only genetic aberration that has implication for MDS diagnosis and classification.

The torrent of novel genetic information is not yet reflected in the way MDS is being diagnosed and classified. The limitations of morphologic classification lead to the recognition of ill-defined entities.^7^ There is an unmet need for laboratory-supportive tools for the diagnosis and classification of morphologically challenging entities.^8^,^9^ The potential of genetic data that can support current diagnostic tools is of special importance when morphologic diagnosis is difficult, inconclusive, or non-reproducible by different morphologists. Newly identified driver mutations are found to be associated with otherwise poorly defined diseases and are likely to be included in future MDS or myeloproliferative neoplasm (MPN) classification. Examples for suggested driver mutations are the SET-binding protein 1 (*SETBP1*) in atypical chronic myeloid leukemia (aCML),^10^,^11^ *SRSF2* in chronic myelomonocytic leukemia (CMML),^12^–^14^ and *CSF3R* in chronic neutrophilic leukemia.^15^,^16^ Another common diagnostic difficulty that can now be overcome using genetic information is the differentiation between primary myelofibrosis and other causes of bone marrow fibrosis. Either calreticulin (*CALR*) mutation or *JAK2* mutation is revealed in most cases of primary myelofibrosis.^17^ It is therefore reasonable to anticipate incorporation of these mutations into future diagnostic and classification criteria.^18^,^19^

In low-risk MDS, criteria for quantitative enumeration of ring sideroblasts are available,^20^ but the techniques require well-trained morphologists and are time-consuming. One may consider that since the *SF3B1* mutation has a positive predictive value for the presence of ring sideroblasts of 97.7%,^21^ this mutation will soon be recognized as supportive to the diagnosis of refractory anemia with ring sideroblasts. Chronic myelomonocytic leukemia (CMML) is currently classified, based on the level of the white blood cell count, into myelodys-plastic and myeloproliferative subcategories. It was recently suggested that such sub-classification could be better defined by specific genetic profiles.^22^,^23^

Understanding of the genetic basis of myeloid malignancies will inevitably lead in the near future to the incorporation of some of the above-mentioned genetic tests into the working schemes for hematologic diagnosis. Potentially, the identification of pre-leukemic mutations in AML^24^ may be the first step towards accomplishing a more challenging task, that of determining the boundaries between high-risk MDS or MPN and AML. In the future, genetics may enable molecular identification of patients in whom leukemic transformation would ensue prior to the appearance of traditional clinical and laboratory signs and symptoms.

### Prognostication: Cytogenetics and Beyond

For many years, cytogenetics has been the only genetic information that influenced clinical decisions in MDS. The international prognostic scores both in their primary and revised versions (IPSS and R-IPSS)^25^,^26^ consider only patients who received supportive therapy and describe the natural history of the disease providing valuable predictions of prognosis. Recent large-scale, comprehensive genetic studies have provided description of the mutation landscape of MDS,^27^–^29^ identification of new mechanisms involved in disease pathogenesis,^30^–^32^ and confirmed that MDS is a stem cell disorder.^18^ Results of such exciting scientific progress are still waiting to be incorporated into daily clinical practice.

Unlike MDS, where cytogenetics allows segregation of patients into categories with a well-defined predicted outcome,^33^ the prognostic value of cytogenetics is much less prominent in CMML^34^–^36^ and primary myelofibrosis (PMF).^37^,^38^ In the absence of a strong cytogenetic influence, the prognostic value of specific mutations is recognized in these diseases. It is not clear whether these mutations have no effect in MDS or their prognostic value in this disease is masked by a strong cytogenetic influence. The *ASXL1* mutation is most dominant in CMML^39^,^40^ and PMF,^41^,^42^ while mutations in other genes may also affect patient prognosis. The interplay between cytogenetic profile and prognostic power of specific mutations is highlighted by the finding that most MDS patients presenting with a complex karyotype also carry a mutation or a deletion in the well-known tumor suppresser gene *TP53*.^43^,^44^ Whether the aberrant karyotype or the *TP53* mutation is responsible for the poor outcome of affected patients is still unknown.

The more we know about the genomic landscape of myeloid malignancies, the more complicated become the combinatorics of potential aberrations to be integrated for prediction of the outcome for a specific patient. Cytogenetics, mutations, micro-deletions, methylation abnormalities, and micro-RNA expression levels are all important players determining patient prognosis. It will therefore be challenging to create an ultimate prognostic model which will be much better than those available today. Physicians should focus on genetic aberrations that can influence therapeutic decisions in every given clinical situation. Examples and suggestions for such an approach are reviewed herein.

## THERAPEUTIC DECISIONS IN THE ERA OF MULTIPLE TREATMENT OPTIONS

In previous years, allogeneic hematopoietic stem cell transplantation (allo-HCT) was the only disease-modifying therapy available for MDS. Allo-HCT is indicated whenever the risk of death or leukemic transformation is projected to be lower following transplant than the equivalent risk anticipated by the Revised International Prognostic Scoring System (R-IPSS).^45^ The International Prognostic Scoring System (IPSS) and R-IPSS considered only patients who received no other therapy than supportive care, but nowadays, due to a tremendous progress in the area of MDS, most patients are candidates for novel disease-modifying drugs (e.g. azacitidine^46^,^47^ and/or lenalidomide^48^–^50^). Retrospective comparisons suggest that allo-HCT may be superior to azacitidine in selected high-risk populations.^51^–^53^ However, patients’ specific consultation must be outlined with caution, because such retrospective data are subject to inherent biases.^54^ Allo-HCT may not be suitable for many AML/MDS patients presenting at older age and with co-morbidities.^55^–^59^ However, with modern reduced-intensity conditioning and supportive care, a large proportion of patients with myeloid diseases are considered fit and can survive through transplant. Many of these potential candidates for allo-HCT are classified as intermediate-risk, and, even when a survival benefit with allo-HCT compared to supportive care is predicted by R-IPSS, the actual clinical debate is whether allo-HCT will lead to an outcome better than that provided by novel therapies. Notably, patients may prefer hypomethylation therapy over transplant in fear of hampered post-transplant quality of life.

### Novel Agents and Associated Dilemmas in Acute and Chronic Leukemia

During the last decades, targeted therapies with tyrosine kinase inhibitors and all trans-retinoic acid have revolutionized the management of chronic myeloid leukemia (CML) and acute promyelocytic leukemia (APL). In 2014, promising results were reported on the use of additional novel targeted agents (e.g. *FLT3-ITD* and *IDH1* inhibitors in AML,^60^,^61^ ibrutinib and ABT-199 in chronic lymphocytic leukemia (CLL)^62^,^63^). Approvals of many of these novel drugs are based on results of phase II studies with short follow-ups, making the incorporation of these agents into clinical algorithms challenging. Targeted therapies aim at specific mutations, but, since mutations in different genes often coexist, identification of the targeted mutation does not guarantee clinical response to its inhibitor. Even if a good response is achieved, its longevity is not known. Predicting the durability of the benefits of targeted therapies is essential, especially when traditional alternatives are associated with high morbidity and mortality rates. Imagine older adults with *FLT3-ITD*-positive AML and normal karyotype or fludarabine-resistant CLL, who responded well to quizartinib or ibrutinib consulting you whether to proceed to allo-HCT. Would your advice be different if a patient diagnosed with Ph+ acute lymphocytic leukemia were consulting you after achieving a good response with dasatinib? Firm clinical data will be available to support the selection of specific targeted therapies over allo-HCT only in another few years when follow-ups mature. However, with the pace that novel targeted therapies enter clinical practice, physicians should come up with rational thoughts guiding the best decisions even when available data are premature. Safety profiles of most targeted drugs are good, and thus they become attractive, which challenges the justification for using high-risk procedures like allo-HCT. Availability of novel agents is increasing, and, for those who achieved good responses, prediction of longevity and potential curability is important, especially for the growing population of older adults fit for reduced-intensity conditioning allo-HCT.

## GENETIC DATA TO SUPPORT THERAPEUTIC DECISIONS IN THE LACK OF CLINICAL EVIDENCE

Researchers, looking for the very first cancer-initiating event, often concentrate on driver mutations that have the power to transform a normal cell into a malignant one. Yet, some available and effective drugs target mutations which lack the capacity to kick off cancer by themselves but are essential for cancer growth and proliferation. Hematological malignancies vary in their genetic complexity. Not surprisingly, CML and APL, the two success stories of targeted therapies, are characterized by a single dominant oncogenic hybrid protein as their driver event. Pathogenesis of most other hematological malignancies is much more complex, with multiple coexisting combinations of genetic aberrations generating signals stimulating progression of the tumor. In AML, nucleophosmin (*NPM1*) and *FLT3* are the two most commonly mutated genes that have been studied extensively. Both have clinical significance, but biological differences between these mutations should be considered which may help outlining a scheme for translating basic genetic information into rational clinical moves. The most important biological difference between *NPM1* and *FLT3* mutations lies in the time point where they occur during leukemogenesis. The *NPM1* mutation is likely to occur very early during the pathogenesis of leukemia. When injected to immunocompromised mice, *NPM1*-mutated leukemic cells can generate leukemia.^64^ The *NPM1* mutation is stable at diagnosis and relapses and is therefore considered a potential marker for minimal residual disease (MRD).^65^ On the contrary, *FLT3-ITD* is recognized as a late event,^66^ may be sub-clonal,^67^ and is therefore not reliable as an MRD marker. Indeed, *NPM1*, but not *FLT3-ITD*, was recognized as a provisional entity in the 2008 World Health Organization classification of myeloid neoplasms.^68^ However, although *FLT3-ITD* has no capacity of *NPM1* mutation to transform a normal hematopoietic cell into a leukemic one, whenever both mutations coexist, the favorable prognostic effect of *NPM1* mutation is overridden by the deleterious prognostic effect of *FLT3-ITD*.^69^,^70^ Similarly, t(8:21) is considered to be an early event which yields a well-characterized oncogenic hybrid protein and is stable to be used as an MRD marker.^71^,^72^ Yet, concomitant *C-kit* or *ASXL1* mutations which are later events may significantly hamper the prognosis.^73^,^74^

The clonal evolution nature of myeloid malignancies^75^–^77^ is well established. Disease progression may be attributed to a late event resulting in a proliferation and/or survival benefit to a sub-clonal population. Targeted therapies that could block such mutation may lead to a tremendous initial clinical response, but one should bear in mind that such response may be short since the therapy is not targeting the primary pathological event. On the contrary, drugs that target the primary event may not be powerful enough to oppose a strong proliferative signal derived from a “second hit” developed in the subpopulation that took over the entire tumor. As there is no reliable mechanism to predict who of the many patients presenting with similar diseases will respond to a specific agent, there is no model forecasting the duration of response in those who achieved a clinically meaningful response.

## NEXT-GENERATION SEQUENCING—DO CLINICIANS NEED SO MUCH INFORMATION?

Genetic profiling of hematological diseases is part of standard assessment. This is particularly significant for acute leukemia, where clinical decisions sometimes depend on the presence or absence of specific mutations.^78^ The development of next-generation sequencing (NGS) techniques allows parallel quantitative sequencing of large numbers of genes at an affordable price.

Next-generation sequencing yields an enormous amount of data which are difficult to interpret, thus requiring skilled bioinformatics expertise. In addition, there is no consensus regarding technical merits of NGS clinical use.^79^ Laboratories performing NGS usually provide a comprehensive report including data on multiple genetic aberrations, and in some cases deep sequencing gives information about sub-clonal mutations.

Clinicians are usually familiar with traditional statistical analysis for prognostic evaluation based only on the most common mutation combinations in AML, leaving most patients in a heterogeneous “intermediate-risk” group.^80^,^81^ There is an unmet need for creating a clinically valid and meaningful “dictionary” for the growing NGS-derived data from private or academic NGS facilities. Clearly, from the clinical perspective not all mutations matter. Mutations with a “weak” prognostic effect are anticipated to be recognized when data from a higher number of analyzed patients become available. Yet, would you recommend your patient spending money on private detailed NGS?

## CLINICAL POTENTIAL OF NEXT-GENERATION SEQUENCING

Genetics is perceived by the majority of hematologists as the most accurate prognostication tool. Next-generation sequencing is therefore acknowledged for high-level prognostication which, unfortunately, we have limited capacity to deal with. However, NGS is much more than a way of identifying all mutations at a reasonable price. This technique provides an opportunity for dynamic prognostication of patients presenting with myeloid malignancies who may experience leukemic transformation. In recent years, sequential genetic evaluation of the same patient demonstrated the dynamics of progression from MDS to secondary AML.^29^,^75^ Preliminary studies suggested that accumulation of sub-clonal mutations identified by sequential meticulous genetic follow-up can point out an evolving leukemic transformation.^29^,^82^ Even mutations which in general have no prognostic value, such as those found in the RAS genes,^83^ may be alarming if they emerge in patients previously tested and found to be negative for these mutations, regardless of R-IPSS classification.^84^ Specific mutations in *ASXL1*, *BCOR*, and *IDH1* are also associated with a pending leukemic transformation.^82^,^85^–^87^ The complexity of leukemia genetics puts an obstacle to revealing the significance of every single mutation for therapeutic decisions. Next-generation sequencing may bring a new dynamic attitude, in which not the presence of a specific mutation but the dynamics and acquisition of new mutations will be of clinical importance. For example, arguments in favor of allo-HCT may sound stronger for patients in whom deterioration toward leukemic transformation can be pre-identified.

## CONCLUSIONS

Techniques for DNA sequencing are rapidly and constantly improving. Clinicians are facing a challenge to employ the new capacities of genetic laboratories to make sure that their patients will benefit most from the ongoing scientific progress. The current review has discussed the contribution of different genetic testing tools to clinical practice. Myeloid malignancies are among the most genetically studied cancerous diseases where genetics is recognized as an important player in diagnosis and/or classification. Our growing understanding of the underlying genetic mechanisms of leukemogenesis may help physicians in daily practice, especially when patients are facing questions where no firm evidence exists to support a specific decision. The explosion of novel targeted agents makes such circumstances frequent. Next-generation sequencing should be considered advantageous, not only because it provides access to a wide spectrum of genetic testing for all patients, but also because it may allow recognition of genetics as a dynamic assessment tool of clonal evolution. It may be suggested that in the future, regardless of the presence of a specific mutation, a pending leukemic transformation may be predicted by sequential monitoring of the same patient for newly acquired mutations. Genetics is about to go beyond prognostication and will soon be strongly incorporated into many aspects of clinical evaluation and therapeutic decisions.

## Abbreviations:

allo-HCT: allogeneic hematopoietic cell transplantation;
AML: acute myeloid leukemia;
APL: acute promyelocytic leukemia;
CLL: chronic lymphocytic leukemia;
CML: chronic myeloid leukemia;
CMML: chronic myelomonocytic leukemia;
IPSS: International Prognostic Scoring System;
MDS: myelodysplastic syndrome;
MPN: myeloproliferative neoplasm;
MRD: minimal residual disease;
NGS: next-generation sequencing;
PMF: primary myelofibrosis;
R-IPSS: Revised International Prognostic Scoring System.
